# Breaking Barriers in Neuro-Oncology: A Scoping Literature Review on Invasive and Non-Invasive Techniques for Blood–Brain Barrier Disruption

**DOI:** 10.3390/cancers16010236

**Published:** 2024-01-04

**Authors:** Miłosz Pinkiewicz, Mateusz Pinkiewicz, Jerzy Walecki, Artur Zaczyński, Michał Zawadzki

**Affiliations:** 1 Faculty of Medicine, Wroclaw Medical University, 50-367 Wrocław, Poland; 2 Department of Diagnostic Imaging, Mazowiecki Regional Hospital in Siedlce, 08-110 Siedlce, Poland; 3 Division of Interventional Neuroradiology, Department of Radiology, The National Medical Institute of the Ministry of the Interior and Administration, 02-507 Warsaw, Poland; 4 Department of Neurosurgery, The National Medical Institute of the Ministry of the Interior and Administration, 02-507 Warsaw, Poland; 5 Department of Radiology, Centre of Postgraduate Medical Education, 01-813 Warsaw, Poland

**Keywords:** blood–brain barrier, blood–tumor barrier, osmotic disruption, laser interstitial thermal therapy, convection-enhanced delivery, focused ultrasound, low-intensity pulsed ultrasound, tumor-treating fields

## Abstract

**Simple Summary:**

Advancements in understanding the blood–brain barrier (BBB) and blood–tumor barrier (BTB) structures emphasize the need to explore functional differences influencing drug distribution to the central nervous system (CNS). Various methods for overcoming the BBB/BTB have been proposed. Osmotic BBB disruption with intra-arterial administration effectively obtains local, clinically relevant concentrations in large brain areas, with an acceptable safety profile in experienced hands. Convection-enhanced delivery allows for homogenous and safe drug delivery to the tumor but demands further technical refinement and clinical evaluation. Emerging therapies like high-intensity focused and low-intensity pulsed ultrasound show great promise in delivering large therapeutic agents to small brain areas. While laser interstitial thermal therapy is effective in ablating local brain tumors, its impact on disrupting the BBB remains inadequately understood. Similarly, tumor-treating fields aid chemotherapy in inducing cycle arrest, but the proof of their ability to disrupt the BBB/BTB has been so far limited to preclinical studies.

**Abstract:**

The blood–brain barrier (BBB) poses a significant challenge to drug delivery for brain tumors, with most chemotherapeutics having limited permeability into non-malignant brain tissue and only restricted access to primary and metastatic brain cancers. Consequently, due to the drug’s inability to effectively penetrate the BBB, outcomes following brain chemotherapy continue to be suboptimal. Several methods to open the BBB and obtain higher drug concentrations in tumors have been proposed, with the selection of the optimal method depending on the size of the targeted tumor volume, the chosen therapeutic agent, and individual patient characteristics. Herein, we aim to comprehensively describe osmotic disruption with intra-arterial drug administration, intrathecal/intraventricular administration, laser interstitial thermal therapy, convection-enhanced delivery, and ultrasound methods, including high-intensity focused and low-intensity ultrasound as well as tumor-treating fields. We explain the scientific concept behind each method, preclinical/clinical research, advantages and disadvantages, indications, and potential avenues for improvement. Given that each method has its limitations, it is unlikely that the future of BBB disruption will rely on a single method but rather on a synergistic effect of a combined approach. Disruption of the BBB with osmotic infusion or high-intensity focused ultrasound, followed by the intra-arterial delivery of drugs, is a promising approach. Real-time monitoring of drug delivery will be necessary for optimal results.

## 1. Introduction

Formed by a monolayer of microvascular endothelial cells cemented by tight junctions, the blood–brain barrier (BBB) serves as the primary site for the blood–central nervous system (CNS) exchange, offering a total exchange area ranging from 12 to 18 m^2^ for the average human adult brain [1,2,3,4]. The BBB tightly regulates the flow of molecules to the CNS, thereby ensuring optimal concentrations of vital substances for neurons and glial cells while shielding the CNS from neurotoxic molecules circulating in the bloodstream [3,4,5]. While substances essential for meeting the metabolic demands of nervous tissue traverse the BBB through various mechanisms, the BBB is characterized by low vascular permeability, effectively preventing the majority of blood-borne molecules, with some notable exceptions such as alcohol or caffeine, from penetrating the brain parenchyma [1,3,6,7]. However, it is crucial to note that the BBB does not represent a uniformly impermeable vascular wall. Numerous studies have showcased variations in BBB permeability within distinct regions of the brain [6,7,8,9]. For instance, research on rats revealed a higher uptake of insulin in the hippocampus compared to the cortex [10]. Moreover, specific brain regions, such as the circumventricular organs and the subependymal zone, exhibit elevated permeability in contrast to the overall lower permeability observed in the rest of the brain [7,11]. Similarly, particular nuclei adjacent to the third and fourth ventricles, including the subfornical organ, area postrema, pineal gland, and median eminence, have vessels with much greater passive permeability [12].

While the highly selective nature of the BBB is essential from an evolutionary standpoint, it is the primary factor restricting effective drug delivery to the brain and the major factor contributing to historically poor outcomes following brain cancer chemotherapy. In spite of significant advances in the field of neuro-oncology, such as the emergence of targeted biologic therapies, the BBB continues to be the main obstacle in improving outcomes following drug administration.

Contrary to a commonly held misconception that small molecules, i.e., <400 Da, readily cross the BBB, histamine, which is only ~100 Da in molecular mass, penetrates the porous capillaries perfusing all peripheral tissues but is prevented from entering the brain or spinal cord by the BBB [1,13]. Given that the size of the majority of chemotherapeutics is in the range of 200–1200 Da, approximately 98% of small molecule drugs and almost all large drugs, such as monoclonal antibodies (mAbs), recombinant proteins, antisense, or gene therapeutics, fail to effectively penetrate the brain parenchyma and therefore exert a therapeutic effect [13,14]. Moreover, even if some drugs with favorable chemical properties, including lipophilic profile, molecular weight <400 Da, and forming less than 8 hydrogen bonds may cross the BBB via lipid-mediated free diffusion, as is the case with temozolomide, achieving effective local concentrations is often impossible since systemically administered drugs have to endure considerable dynamic forces within the brain interstitium induced by cerebrospinal fluid (CSF) flow, intratumoral edema, and pressure effects related to tumor mass [15,16,17,18,19]. These factors explain why temozolomide reaches only 20–30% of plasma concentrations in the CNS [20,21].

As the brain tumor progresses, the BBB starts to undergo architectural changes, leading to a structure known as the blood–tumor barrier (BTB). Although more permeable than a healthy BBB, clinical experiences have demonstrated that the BTB’s permeability to both small and large molecules is, in fact, highly heterogeneous [22]. Moreover, due to the BTB’s uneven perfusion, drug delivery is far from optimal, mirroring the challenges observed with the BBB [22]. However, brain tumors are not the only pathology that disrupts the BBB as multiple neurological diseases, including acute and chronic cerebral ischemia, brain trauma, multiple sclerosis, and brain infections, induce cellular damage that compromises the BBB’s integrity [23].

Considering the evident failure of transporting drugs across the BBB/BTB, researchers have been strongly driven to establish safe and effective ways of circumventing, disrupting, or manipulating the BBB/BTB to improve patient outcomes after chemotherapy [24].

Thanks to the more elaborate understanding of the BBB and a rising sense of frustration with poor outcomes following brain cancer treatment, we have been witnessing a concerted effort dedicated to the development of various invasive and non-invasive methods that can improve drug delivery through BBB disruption.

This scoping literature review provides a comprehensive examination of both invasive and non-invasive methods of BBB and BTB disruption. It delves into the underlying concepts, presents insights from preclinical and clinical experiences, and discusses the advantages, disadvantages, indications, and areas requiring further optimization. Notably, unlike other comprehensive reviews, this work distinguishes itself by offering detailed descriptions of neurosurgical and neurointerventional techniques without delving into pharmacological solutions for enhancing drug penetration across the BBB/BTB. Additionally, we explore crucial findings concerning the structural aspects of the BBB/BTB and their implications for the clinical work.

The following parts of the review go over the BBB and the BTB structure both in metastatic and primary brain tumors. The middle part of the review describes invasive methods, such as osmotic disruption, intrathecal/intraventricular administration, laser interstitial thermal therapy (LITT), and convection-enhanced delivery (CED), with a paragraph dedicated to optimizing osmotic BBB disruption, followed by the characterization of non-invasive methods of BBB disruption, including high-intensity focused and low-intensity pulsed ultrasound and tumor-treating fields (TTFields). Subsequently, we highlight potential avenues for further research and improvement and conclude with final comments.

## 2. Materials and Methods

### 2.1. Search Strategy and Selection Criteria

A literature search was conducted in July 2023 in the Medline/PubMed, Cochrane, Google Scholar, Scielo, and PEDro databases for entries in English from inception to search date. In short, the search strategy used the following keywords (in the given order): “Blood-brain-barrier”, “Blood-tumor-barrier”, “Osmotic disruption”, “Laser interstitial thermal therapy”, “Convection-enhanced delivery”, “High-intensity focused ultrasound”, “Low-intensity pulsed ultrasound”, “Tumor-treating fields”.

The systematic review followed the recommendations of the Preferred Reporting Items for Systematic Reviews and Meta-Analyses (PRISMA). The protocol has not been registered.

### 2.2. Study Selection and Data Extraction

Non-peer-reviewed papers and records not available in the full text have not been included. Also, studies were excluded if there was incomplete or missing data. We have excluded conference abstracts. Five independent reviewers (Mi.Pinkiewicz, Ma. Pinkiewicz, Walecki, Zaczyński, and Zawadzki) performed a primary title and abstract screening and extracted full data from eligible articles following the PRISMA extension for scoping reviews (PRISMA-ScR) guidelines. We have chosen articles for inclusion on the grounds of study quality and design. We have included studies focusing on the BBB structure, BTB structure, osmotic blood–brain barrier disruption, imaging advancements for intra-arterial cerebral infusions, ultrasound approaches, tumor-treating fields, convection-enhanced drug delivery, and laser interstitial thermal therapy. We have reviewed and included selected preclinical and clinical studies concerning these methods of overcoming BBB. The judgments concerning the risk of bias were formed by two reviewers and subsequently double-checked by another two reviewers. Discrepancies in data extraction and synthesis were resolved by consensus decision of all reviewers.

## 3. Results

**Figure 1:**
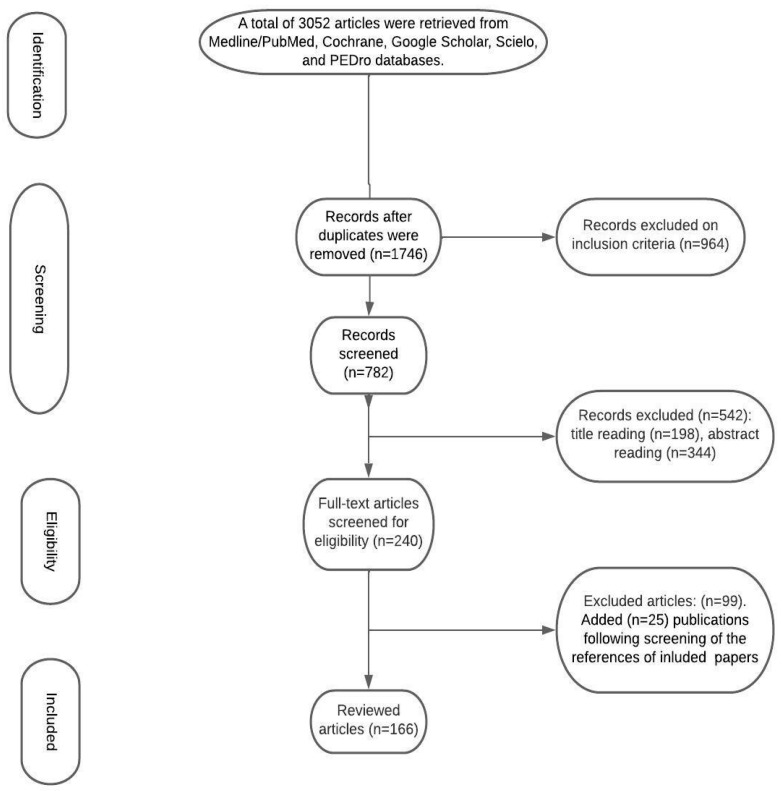
Flow diagram demonstrating the process of article selection.

The initial search of the Medline/PubMed, Cochrane, Google Scholar, Scielo, and PEDro databases using the aforementioned keywords resulted in the retrieval of 3052 papers. Screening for duplicates and their removal resulted in a total of 1746 articles. Subsequently, we excluded 964 articles due to the language and study design. The titles or abstracts of 782 articles were screened, which obtained 240 papers that did not meet any of the exclusion criteria. After a full-text evaluation of 240 papers, we excluded 99 papers and added 25 publications following a screening of the relevant papers. This led to the inclusion of 166 articles. Figure 1 demonstrates our process for article selection.

### 3.1. The BBB’s Structure

**Figure 2:**
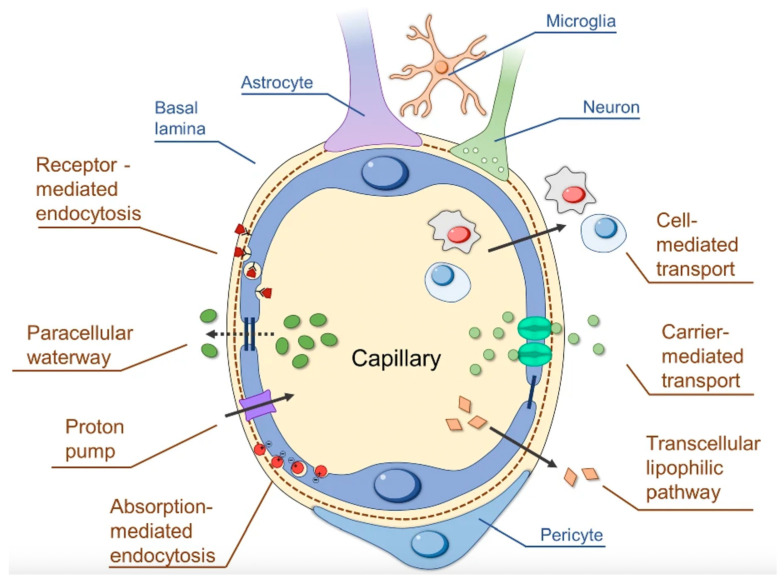
Depicts the BBB’s structure and transport mechanisms. The BBB’s structure consists of brain microvessels involving pericytes, endothelial cells, astrocytes, and neurons. Mechanisms for transport across the BBB include the following: diffusion (transcellular lipophilic pathway), carrier-mediated transport (CMT), receptor-mediated endocytosis (RME), absorption-mediated endocytosis (AME), proton pump, cell-mediated transport, and paracellular waterway. Reused from Mitusova et al. [29].

**Figure 3:**
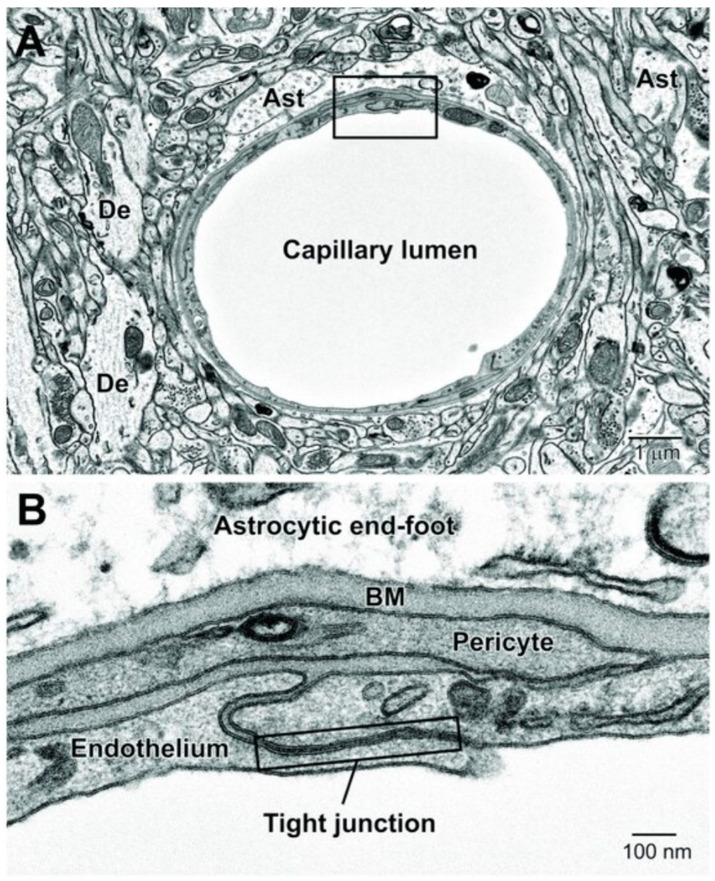
The BBB and neurovascular unit. (**A**) In a cross-sectional view of a mouse cortical capillary, the endothelium appears highly attenuated, featuring small branches of pericytes on its abluminal surface. (**B**) The BBB is composed of the capillary endothelial cells with their tight junctions and underlying pericytes, both surrounded by an amorphous basement membrane (BM) and astrocytic end-feet. Ast, astrocyte; De, dendrite. Reused from Nahirney et al. [38].

Anatomically, the BBB is found at the level of the brain microvascular network, consisting of capillaries, arterioles, and venules [1,25]. The barrier itself is composed of microvascular endothelial cells (ECs) lining the cerebral capillaries, surrounded by brain pericytes embedded in a shared basement membrane (BM) and encased by astrocytic end-feet (Figure 2) [1,3,26,27]. Junctional complexes, including tight junctions (TJs) and adherens junctions (AJs), connect brain ECs, creating a layer of polarized cells with two different sides—luminal and abluminal—each side having distinct lipids and proteins responsible for the blockage of the paracellular transport of small and large water-soluble molecules into the brain, with the exception of minuscule or gaseous molecules such as water and carbon dioxide (Figure 3) [1,3,26,27]. Situated on the apical membrane of brain ECs, TJs are composed of integral membrane proteins, i.e., claudin, occludin, junction adhesion molecules (JAMs), such as JAM-A, -B, and -C as well as various cytoplasmic accessory proteins, including Zonula occludens-1, -2, -3 (ZO-1, ZO-2, ZO-3) and cingulin [1,3,26,27]. Being the first TJ protein discovered, occludin limits small molecules from passing through the BBB, whereas JAM-A serves as a barrier against molecules larger than 4 kDa, preserving BBB impermeability even in cases when claudin proteins are deficient [1,3,26,27,28,29]. AJs are located below the TJs and are made of a cadherin–catenin complex and its associated proteins [1,27,30]. Besides regulating paracellular permeability, AJs maintain the BBB structural integrity and are essential for the appropriate assembly of proteins of TJs [1,3,26,27,28,29].

Surrounding approximately 30% of the endothelial layer, pericytes affect the passage of molecules into the brain through two mechanisms [31,32,33]. Firstly, they maintain BBB integrity via the inhibition of transcytosis and induction of endothelial cell expression of tight junction proteins [32,33]. Secondly, pericytes exert control over vessel diameter and, thereby, cerebral blood flow [32,33,34].

The passage of molecules into the brain parenchyma, i.e., the amount of substance per unit gram of brain tissue, is dependent on the permeability characteristic of the BBB and the surface area of the BBB available for exchange, which together form the permeability surface product [32,33,35]. Alterations in cerebral blood flow have insignificant effects on the absolute amounts of circulating substances entering the brain if a molecule is associated with poor BBB permeability [33]. On the contrary, if the substance is very BBB permeable by its essence or the BBB becomes highly permeable due to pathology, cerebral blood flow starts to become a crucial factor [33]. Thus, through the regulation of the BBB surface and cerebral blood flow, pericytes regulate barrier function in a region-specific manner [33].

The BM is a thin layer of extracellular matrix consisting of four major proteins: collagen IV, laminin, nidogen, and perlecan [36]. Linked with the BM via their end feet, astrocytes, also known as astroglia, form a complex network surrounding the capillaries and are responsible for the induction and maintenance of various BBB properties, such as the polarized expression of transporters on endothelial membranes. However, although astrocytes have many functions in the CNS, such as clearing waste, tuning brain blood flow, regulating vascular function, ion hemostasis, and balancing neuroimmune responses, specific mechanisms by which astrocytes contribute to BBB function remain unknown [1,3,27,37].

The restrictive properties of the BBB were largely unknown until the pioneering electron microscopy work by Brightman, Reese, and Karnovsky demonstrated that, unlike other vascular ECs lining peripheral blood vessels, brain microvascular ECs have unique morphological, structural, and functional features, including the presence of the aforementioned protein junctions (TJs and AJs), low degrees of leukocyte adhesion molecules that effectively impede the entry of the number of immune cells or the absence of fenestrations, also known as small transcellular pores, which restrict free diffusion and the rapid exchange of molecules between tissues and blood [1,27,39,40]. Moreover, given that brain ECs have a net negative surface charge, they do not accept negatively charged compounds [1,26].

Although under physiological conditions, molecules may cross the BBB via the diffusion (transcellular lipophilic pathway), carrier-mediated transport (CMT), receptor-mediated endocytosis (RME), absorption-mediated endocytosis (AME), proton pump, cell-mediated transport, and paracellular waterway (Figure 2), brain ECs are characterized by remarkably low levels of vesicle trafficking due to the high transendothelial electrical resistance, significantly limiting transcellular transport or transcytosis [1,26]. While the specialized TJs have the dominant role in ensuring BBB impermeability, recent investigations have demonstrated that active suppression of transcytosis is paramount for the BBB’s integrity, and it is dynamically regulated during development and disease [41,42].

Brain ECs express specific transporters for regulating the inflow and outflow of desired substrates. Solute carriers (SLC), the largest family of transmembrane transporters, with researchers identifying approximately 287 SLC genes in the brain to date, facilitate the transcellular transport of specific substances like amino acids, carbohydrates, and hormones across the cell membrane [1,43,44]. Likewise, various efflux transporters can be found on the surface of CNS endothelial cells, such as the members of the ATP-binding cassette (ABC) transporter family, including P-glycoprotein (Pgp), breast cancer resistance protein, and multidrug resistance-associated proteins [1,43,44,45]. These transporters export metabolites and xenobiotics, including most anticancer drugs from the brain and endothelium into the bloodstream.

The BBB functions as a part of a larger neurovascular unit (NVU), which is comprised of microglial and neuronal cells in addition to microvascular ECs, the capillary BM, astrocytes, and pericytes (Figure 3) [1,27,28,46]. The cross-talk between these distinct cellular elements of the NVU is essential for CNS homeostasis [1,27,38,46]

### 3.2. The BTB

The concept of the BTB emerged in scientific literature during the latter part of the 20th century as a result of observations that specific types of brain metastasis, including those originating from breast, lung, or melanoma, as well as primary brain tumors, such as gliomas, provoke numerous alterations in the BBB, resulting in a more permeable structure known as the BTB [17,22,47,48].

**Figure 4:**
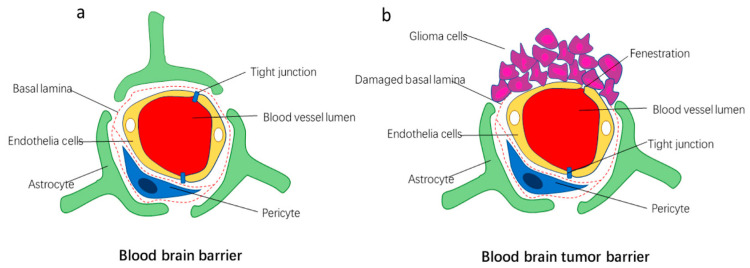
Differences in structure between the BBB (**a**) and BTB (**b**). The BBB consists of a monolayer of non-fenestrated endothelial cells connected by tight junctions. It involves interaction with astrocyte foot processes and pericytes, along with the presence of a functional basement membrane. As the tumor progresses, invasive cells provoke architectural changes in the normal brain vasculature, resulting in fenestrated endothelial cells, disrupted basement membrane (basal lamina), and unattached astrocytes and pericytes. The BTB is also characterized by increased pinocytic activity. Reused from Qiu et al. [48].

Various factors, such as abnormal tumor vasculature, changes in the expression of junctional proteins, inflammatory response, and loss of protein components, such as laminin α2 in the astrocytic basement membrane, collectively contribute to the formation of the BTB, which is characterized by abnormal pericyte distribution as well as loss of astrocytic end-feet, tight junctions, and neuronal connections [17,22,47,48,49]. Consequently, three different microvessels can be found in the BTB; continuous capillaries, fenestrated capillaries, and capillaries possessing inter-endothelial gaps [24] (Figure 4).

Given their proximity and infiltration of brain tissue, primary brain cancers tend to have a higher influence on the structure of the BBB than metastatic brain cancers [17,22,47,48,49].

While our understanding of the BBB/BTB has improved, the majority of findings come from studies conducted on rodent models. There are still many aspects of BTB formation and function that remain unclear or not fully understood, highlighting the need for further research.

### 3.3. BTB in Metastatic Cancer

Brain metastasis occurs despite the fact that the BBB is impermeable to cancerous cells. This phenomenon could be attributed to the partial blood–brain barrier disruption (BBBD) or transendothelial migration of tumor cells, similar to the transendothelial migration of leukocytes, i.e., rolling, adhesion, and diapedesis [1,50]. According to one study, the opening of endothelial TJs in metastatic adenocarcinoma could be potentially attributed to the loss of the 55 kDa occludin expression in the brain microvessels [51]. Similarly, studies highlight the potential role of aquaporin-4 (AQP4) in BBB disruption, reporting that AQP4 is extensively up-regulated in metastatic adenocarcinoma [1].

Although animal models demonstrated that metastatic lesions have an elevated BTB permeability, it is highly heterogeneous, with only a fraction having sufficient permeability to induce drug responses [1,17,22,52,53,54,55,56,57,58,59,60,61].

Using two models of brain metastases of breast cancer, Lockman et al. reported cytotoxic concentrations of paclitaxel only in a subset (<10%) of the leakiest brain metastases where drug concentration exceeded 1000 ng/g [19]. Even in brain metastases considered most leaky (~33-fold increase), permeability was still <12% of that in a peripheral breast tumor [52]. Similarly discouraging results were reported using vinorelbine, with the authors reporting median brain metastasis drug exposure being fourfold greater than normal brain, yet only ~8% of non-barrier systemic metastases [53]. The heterogeneous distribution of the drug observed in animal models has also been demonstrated in clinical studies, with Morikawa et al. reporting significantly varying concentrations of capecitabine and lapatinib in breast cancer brain metastases, ranging from 1.0 µM to 63 µM [22,60]. Despite the increased permeability of the BTB compared to the BBB, the delivery of conventional therapeutics, molecularly targeted inhibitors, and monoclonal antibodies and antibody-drug conjugates (ADCs) to the brain remains a significant challenge [1,17,22,52,53,54,55,56,57,58,59,60,61]. Likewise, established chemotherapeutic agents such as paclitaxel, doxorubicin, vinorelbine, and temozolomide, as well as molecularly targeted small-molecule inhibitors like gefitinib and lapatinib, have demonstrated limited ability to attain therapeutically effective concentrations within the brain tumors [1,17,22,53,62]. Similarly, monoclonal antibodies and ADCs, such as trastuzumab and ado-trastuzumab emtansine (T-DM1), fail to reach metastatic lesions in optimal amounts [17,22,55,56,58,61].

### 3.4. BTB in Gliomas

The BTB in gliomas presents unique characteristics compared to the healthy BBB [17,22,63,64,65,66]. Gliomas exhibit aberrant pericyte distribution, loss of astrocytic end-feet and neuronal connections, as well as abnormal blood vessel formation provoked by chaotic and disorganized angiogenesis [22,64,65,66,67]. Invading glioma cells disrupt astrocytic end-feet, leading to the formation of highly permeable and leaky blood vessels with irregular structure and function [22,64,65,66,67]. The integrity of TJs is compromised, resulting in increased gaps and fenestrations that allow for enhanced permeability of molecules into the tumor tissue [1,22,64,65,66,67]. Moreover, claudin-1 expression is lost in microvessels, while claudin-5 and occludin are substantially down-regulated [1,68]. Like brain metastasis, the significant up-regulation of AQP4 potentially accounts for the hallmark of malignant tumors: cerebral edema [1,69]. As a result of these changes, gliomas exhibit a permeable BTB, which, due to gadolinium-based contrast agents diffusing out of the vessel lumen and accumulating within the extravascular extracellular space, can be observed as contrast-enhancing hyperintense regions on T1-weighted (T1W) on magnetic resonance imaging (MRI) [70,71]. However, it is crucial to emphasize that increased permeability will only be seen in specific parts of high-grade gliomas (HGG), whereas low-grade lesions have almost fully functional BBBs [71,72].

Various MRI and positron emission tomography (PET) studies demonstrated HGG regions with varying degrees of BBB disruption and vascular permeability [1,17,22,70,71]. In general, the tumor core tends to have higher leakiness when compared with the peritumoral region and the surrounding brain microenvironment, which may maintain an almost fully functional BBB [22]. HGG, as well as the majority of other malignant tumors, are highly infiltrative in nature, with tumor cells extending beyond the primary tumor mass and even beyond the surrounding edema depicted by the FLAIR sequence on MRI scan [73,74]. These distally located tumor areas with an intact BBB shield invasive glioblastoma stem cells (GSCs), which, due to significant levels of transcriptional variability and metabolic flexibility, demonstrate remarkable adaptability properties, making them a primary driver of GBM recurrence and treatment resistance [73]. These findings unequivocally refute the common belief that the BBB is uniformly disrupted in gliomas [71]. With evidence clearly showing that only central tumor regions may benefit from increased drug accumulation due to a “leaky” BTB, it is clear that as long as drugs fail to accumulate in peripheral glioma regions surrounded by an intact BBB, there is no possibility of improving chemotherapeutic outcomes in glioma patients [75].

### 3.5. Disruption of the BBB/BTB

Given that the BBB/BTB makes it impossible to obtain homogenous and high drug concentrations necessary to induce clinically relevant anti-tumor responses, extensive efforts have been dedicated to exploring methods of disrupting the BBB/BTB. Since BBB/BTB’s impermeability is largely based on the presence of TJs, the majority of these approaches, aside from intrathecal/intraventricular administration, aim to disrupt these junctions, either chemically, mechanically, or through various biological pathways to provoke transient BBB/BTB opening.

The field of BBB/BTB disruption has a rich history dating back to the previous century, with methods such as osmotic BBB/BTB disruption or CED having been extensively studied. In contrast, techniques like TTFields, high-intensity focused ultrasound, and low-intensity pulsed ultrasound, though promising, have garnered less attention due to their relative novelty. Similarly, LITT, although described already in the 1980s, has been largely overlooked until the development of spatial and temporal magnetic resonance (MR) thermometry. Moreover, the relative disparity in research quantity may be attributed not only to historical precedence but also to the varying levels of interest that certain methods have generated among researchers, potentially influenced by recent successes or technological advancements. In the subsequent sections of this review, we will delve into the comprehensive exploration of the aforementioned strategies.

### 3.6. Osmotic BBB/BTB Disruption

The principles of osmotic BBBD stem from the works of Stanley Rapaport, who demonstrated that the injection of hyperosmolar agents in a flow rate sufficient to allow a complete filling of the vessel without producing significant reflux in the common carotid artery leads to the reversible dehydration of brain ECs and subsequent disruption of the TJs [74,76,77,78,79]. With animal studies demonstrating osmotic BBBD’s potential to enhance the delivery of chemotherapeutic agents by up to 90-fold, Neuwelt et al. went on to prove that BBBD increased concentrations of methotrexate in patients with CNS lymphoma, reporting successful and durable tumor control [80,81]. Since then, multiple studies have provided evidence that osmotic BBBD increases maximal peak concentration as well as AUC (the concentration of the drug according to the time) of the administered molecule, with the intra-arterial infusion of 1.37 mmol/L mannitol (25%, 10 mL) being most commonly reported [74,81,82,83,84,85,86]. Moreover, osmotic BBBD can disrupt the entire vascular distribution supplying the tumor and surrounding brain [83]. This allows the chemotherapeutic agent to effectively target infiltrative gliomas or multiple tumor foci commonly seen in breast or lung tumor metastasis [83]. However, the degree of mannitol-induced BBB opening may vary depending on the brain region. A study conducted on rats found that the cortex and midbrain had higher sucrose permeability than either the cerebellum or brainstem after mannitol-induced BBB disruption, although to date no studies have evaluated these findings in clinical settings [87].

By offering longer tumor cell exposure to higher concentrations of the administered therapeutics, the osmotic disruption could potentially help overcome the “sink effect” and provide more uniform drug delivery to the entire CNS vascular territory, including tumor edges [74,88,89,90,91,92]. Introduced by Neuwel et al., the “sink effect” describes the phenomenon where chemotherapy agents that cross the CNS and penetrate the tumor face preferential accumulation and concentration in areas of tumor necrosis [74,88,89,90,91,92]. Tumor necrotic areas act as “sinks”, attracting and trapping the administered chemotherapeutics. As a consequence, the peripheral areas of the tumor, including the highly proliferative tumor edges with neoplastic cells, receive lower drug concentrations [74,88,89,90,91,92]. The uneven distribution of the drug throughout the tumor mass significantly limits the efficacy of the treatment [74]. Sato et al. conducted in vivo experiments demonstrating a significant increase in permeability at the edge of the tumor following osmotic BBBD, thereby proving that the sink effect can be successfully overcome [74,92].

Osmotic BBBD has been primarily evaluated in combination with intra-arterial delivery (IA) due to their synergistic effect.

IA administration can deliver over 300 times higher local concentrations of chemotherapeutics to brain tumors when compared to intravenous administration [93]. According to PET measurements, IA leads to a 50-fold increase in brain tumor tissue concentrations when compared to IV injections [94]. When combined with osmotic BBBD, IA delivery can further increase drug exposure by up to 100-fold [95]. Although it has been long ago recognized that the combination of osmotic BBBD with IA delivery can amplify the first pass-through effect in the brain, leading to heightened maximal peak concentration and an increased AUC of the administered molecule, early experiences were associated with considerable neurotoxicity [96,97]. However, significant advancements in catheter technology, such as dual lumen balloons, which allow for modification of blood flow in distal brain vessels to an unprecedented degree, and microcatheters, which together with modern-day angiography, permit for precise and selective targeting of small, distally located vessels supplying the tumor, have led to the refinement of this concept, resulting in the technique known as a super-selective intra-cerebral infusion (SIACI) [97,98,99]. Compared to unselective administration, where a catheter is placed in the vertebral or internal carotid artery, leading to toxicity to non-target tissues such as the eye and normal brain parenchyma, and suboptimal concentrations in the tumor and cerebrospinal fluid, SIACI offers various advantages [97,98,99]. It allows for the precise targeting of cerebral vessels, providing the dual benefits of dose intensification and reduction in neurotoxicity [98,99]. Moreover, SIACI offers a more focused BBBD, making it especially beneficial for nodular lesions, i.e., with a dominant contrast-enhancing lesion and limited surrounding edema volume, where there is no need for the disruption of larger vasculature areas [97,98,99].

Nevertheless, given that SIACI does not ensure a higher drug level in the corresponding brain region as heterogeneous drug delivery may still occur, Gobin et al. introduced a concept of regional dosage based on the vascular territories using a spatial dose fractionation algorithm with a pulsatile delivery method, minimizing neurotoxicity [98,99,100].

Osmotic BBBD with IA delivery can potentially lead to transient cerebral edema due to increased bulk fluid influx. Moreover, it can cause an increase in the influx of molecular compounds, consequently leading to neurological toxicity, aphasia, and hemiparesis. However, extensive research has demonstrated the potential for osmotic BBBD with IA delivery to be both safe and therapeutically valuable, particularly in experienced hands, with severe long-term complications being rare [74,98,99,101].

The Sherbrooke experience involving 319 recurrent glioblastoma patients who underwent IA chemotherapy of carboplatin alone or combined with melphalan or etoposide following osmotic BBBD reported a radiological response in 48% of patients (response assessment in neuro-oncology (RANO) criteria). The authors reported an overall per-procedural seizure incidence of 2% and a total symptomatic vascular complication rate of 0.75% [74]. A recent large retrospective study of patients with primary and metastatic brain tumors who received IA chemotherapy with osmotic BBBD reported major and minor procedural complications in 27 (0.70%) and 289 (7.53%) of 3837 procedures, respectively [101]. In contrast, among the patients who did not undergo osmotic BBBD, the authors observed major and minor procedural complications in 12 (1%) and 41 (3.72%) out of 1102 (1%) IA procedures, respectively [101]. Given that osmotic BBBD with IA delivery carries the risk of focal seizures and vaso-vagal response with bradycardia and hypotension, the Sherbrook experience authors recommend the administration of the following drugs just prior to the disruption: Diazepam 0.2 mg/kg IV (maximum dose = 10 mg), and Atropine IV, titrated to increase heart rate 10–20% from baseline (0.5–1 mg) [74].

Although the lack of phase III clinical trials prevents any definitive statement of safety and efficacy of IA delivery of chemotherapeutics using osmotic BBBD, single-phase 1/phase 2 studies evaluating various chemotherapeutics in patients with glioblastomas and metastatic brain cancers reported encouraging safety and efficacy profiles [97,98,101,102,103]. In 16 patients, Chakraborty et al. have demonstrated that a single IA administration of bevacizumab at 15 mg/kg after osmotic BBBD with mannitol allowed obtaining similar or better progression-free survival in comparison to a biweekly IV infusion of bevacizumab at 10 mg/kg [104]. A study of 23 patients with newly diagnosed glioblastoma (predominantly IDH wild-type) undergoing repeat SIACI of bevacizumab with osmotic BBBD reported a median overall survival of 23.1 months, while 2- and 3-year survival probabilities were 45.0% (95% CI 22.3–65.3) and 32.1% (95% CI 12.5–53.8), respectively, providing foundations for an ongoing phase III clinical trial (NCT05271240) aiming to see if repeated SIACI of bevacizumab after BBBD with mannitol has a long-term therapeutic effect [103].

Despite the extensive use of osmotic BBBD, involving over 4200 procedures across multiple centers and more than 400 patients, there is no consensus regarding the maximum permeability effect or the duration of BBB disruption after mannitol administration [97,105]. Siegal et al. reported that the maximum effect in humans lasts up to 40 min, followed by a rapid decline in permeability, with the normal threshold restored within 6 to 8 h after osmotic disruption [105]. Conversely, Zünkeler et al., using rubidium-82 to measure blood-to-tissue influx, estimated a mean half-time of only 8 min for the return of permeability to almost baseline values in the normal brain [106]. These differences may be attributable to the use of distinct anesthetic agents, which are known to alter cerebral blood flow, varying duration and rate of infusion of mannitol, the tracers, and techniques used to evaluate increased permeability, or the physiological parameters such as blood pressure, heart rate, and PaCO_2_ [105].

Likewise, the optimal dose and infusion time of mannitol, two crucial factors deciding on the extent and duration of osmotic BBBD, lack a universally agreed-upon standard due to the significant influence of individual factors, hindering the establishment of a standardized approach that ensures the maximum permeability effect. Relying on selective delivery with microcatheters, Burkhardt et al. utilized a mannitol infusion rate of 0.083 mL/s for 120 s with SIACI, while Siegal et al. employed infusion rates ranging from 3 to 11 mL/s over a 30 s period using unselective administration with diagnostic catheters placed into the internal carotid or vertebral artery [98,105]. Fortin et al. used iodinated contrast injection and fluoroscopy to determine the individual rate of infusion (the rate that will fill the entire vessel distribution without producing significant reflux in the common carotid artery), which most often is equal to 3 and 6 cc/s × 30 s [74]. Although establishing an optimal infusion time and dose is unlikely due to the significant role of individual factors and distinct treatment goals, proposing standardized guidelines or protocols for dosing would be propitious to guide the practice of osmotic BBB disruption.

### 3.7. Optimizing Osmotic BBBD with Modern Imaging

Imaging modalities have a crucial role in the outcome of IA delivery of therapeutics combined with osmotic BBBD as they allow for the localization of exact vascular tumor supply and guide the selection of the most appropriate vessels [99]. Moreover, given that osmotic BBBD is affected by a number of factors, with the degree of the opening varying significantly, imaging could provide valuable feedback on the extent and exact location of the disrupted BBB, allowing operators to introduce necessary changes in the location of the catheter, infusion rate, or the dose of the osmotic agent to obtain an optimal outcome [107].

Osmotic BBBD with IA delivery is conducted in an angio-suit where digital subtraction angiography (DSA) is used to guide the microcatheter to the most distal intracranial arterial pedicles supplying the tumor to disrupt the BBB and deliver the therapeutic agent [99]. On DSA, BBB disruption is visualized as a contrast blush and early venous enhancement [99].

**Figure 5:**
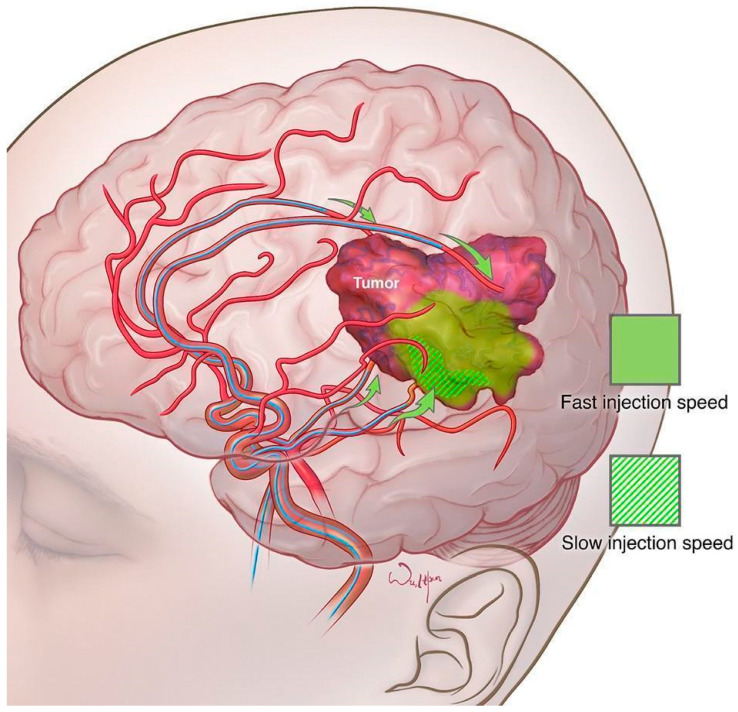
The difference between fast and slow infusion rates during SIACI and their impact on drug delivery. Courtesy of the Society of Image-guided Neurointerventions (SIGN).

Although a golden standard, DSA may be ineffective at the visualization of the smallest intracranial vessels, considering the low sensitivity of contrast agents [97,99,108]. In comparison, MRI contrast agents have a high sensitivity that permits the localization of the smallest concentrations of contrast, especially at the microcirculation level [108]. Consequently, researchers have explored the idea of using an emerging real-time MRI technology for intracerebral chemotherapy delivery with osmotic BBBD, as it could improve the visualization of the region of the brain parenchyma served by the catheter, which is often highly dynamic and subject to variations [108,109]. Moreover, thanks to the MRI guidance, it would be easier to precisely adjust the catheter tip, permitting the operator to deliver the therapy to a selected region of the brain with high accuracy and reproducibility as well as guarantee adequate infusion rate, which is known to have a significant impact on the final outcome of the delivery (Figure 5) [97,108,109].

Last but not least, by providing a precise and immediate view of how the contrast agent, e.g., gadolinium spreads in the brain, neurointerventionalists could adjust the dose of mannitol and the infused therapeutic agent more accurately, reducing the risk of side effects from unselective disruption [97,108,109]. Although real-time MRI is still in development and far from conventional practice, a study assessing the IA delivery route of stem cells labeled with SPION particles (Molday, BioPal, Worcester, MA, USA) in an animal model found that dynamic, real-time MRI monitoring during cell infusion allowed to visualize the process of cell entrapment in the brain, thereby allowing for early intervention should any excessive accumulation and blockage of cerebral flow occur [109]. Following these results, a clinical study by Zawadzki et al. performed three selective IA deliveries of bevacizumab after osmotic BBBD under real-time MRI guidance in an angio-MRI hybrid room [108]. The authors reported that real-time visualization allowed them to optimize drug delivery by adjusting the microcatheter position and infusion rate based on the overlaid tumor area and the calculated degree of overlap [108].

Another intriguing approach for real-time monitoring of the osmotic BBBD is the use of direct-current electroencephalography (DC-EEG), as demonstrated by Kiviniemi et al. [110]. This method capitalizes on the measurable electrical signals at the scalp, which reflect the mV-level potential difference maintained by the intact BBB between blood and brain tissue [110]. In a study monitoring 47 consecutive BBB disruption treatments in nine PCNSL patients, the authors observed pronounced shifts in DC-EEG signals that correlated with the opening and sealing of the BBB [110]. While further research is needed, these findings suggest that DC-EEG holds promise as a real-time monitoring tool for assessing BBB disruption and enhancing drug delivery.

**Figure 6:**
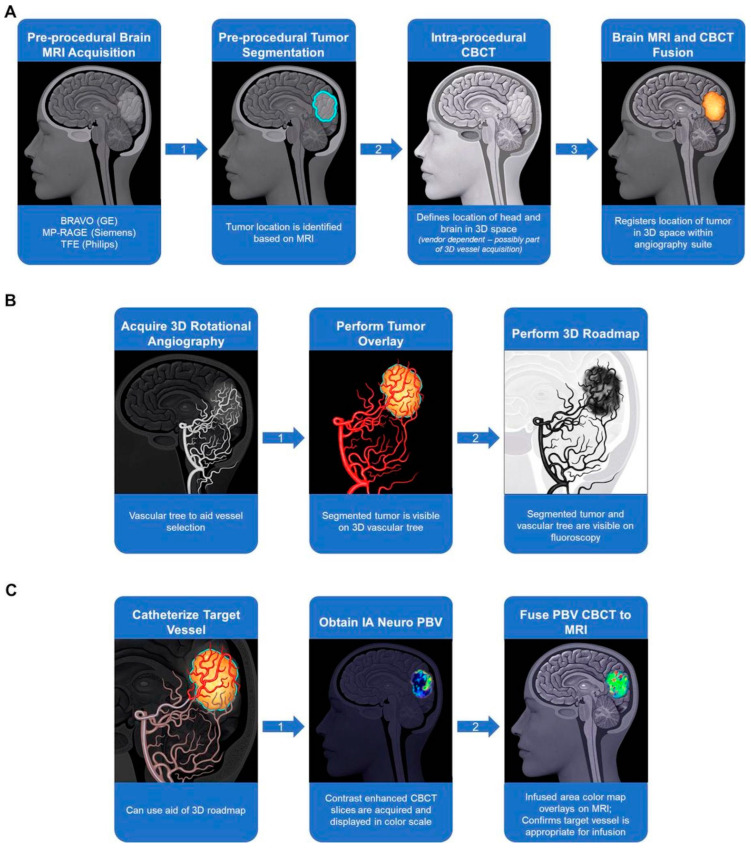
Demonstrates the perfusion-guided endovascular super-selective intra-arterial infusion workflow proposed by Chen et al.: (**A**) Alignment of patient position with MRI. (**B**) Three-dimensional (3D) overlay for vessel selection. (**C**) Integration of parenchymal blood volume (PBV) with MRI. Reused with permission from Chen et al. [111].

Considering the highly variable vascularity of glioblastoma and the fact that it may be impossible to precisely locate the exact vascular supply of GBM with conventional angiography, Chen et al. recommended merging preoperative anatomic MRI images with real-time perfusion images from super-selective injection during angiography (Figure 6) [111]. The authors could accurately locate the vascular supply to brain tumors and facilitate subsequent catheter delivery of the therapeutic agent [111]. This promising method of improving the precision of IA delivery and subsequently verifying that the perfused territory overlaps the tumor volume will be further evaluated in an ongoing clinical trial (NCT03896568) [111].

### 3.8. Intrathecal/Intraventricular Administration

Intrathecal administration involves the direct injection of drugs into either the spinal canal or the subarachnoid space, while the intraventricular method delivers drugs directly into the ventricular system [112,113,114]. In both approaches, the drugs gain access to the cerebrospinal fluid and diffuse across the brain–CSF barrier, allowing for potential therapeutic effects within the CNS [112,113,114].

Intrathecal administration continues to be a mainstay therapy for patients suffering from neoplastic meningitis and has been used in patients with human epidermal growth factor receptor 2-positive (HER2-positive) breast cancer leptomeningeal disease (LMD) and CNS lymphoma [112,113].

The phase II trial of intrathecal trastuzumab (150 mg once weekly) in 19 patients with HER2+ BC with LM demonstrated a median OS of 7.9 months [114]. Similarly, a prospective study of 26 patients with human epidermal growth factor receptor 2-positive (HER2-positive) leptomeningeal disease (LMD) treated with 80 mg of intrathecal trastuzumab has reported an OS of 10.5 months [115]. Although intrathecal administration is inherently associated with potential complications, such as drug-induced aseptic meningitis (DIAM) and infection of the reservoir through which the agents are administered, these two trials did report a favorable safety profile [114,115].

As much as intraventricular and intrathecal administrations may improve drug concentrations in superficially located pathologies, these methods are associated with limited drug distribution within the brain parenchyma, making it challenging to achieve therapeutic levels in deeply located tumors. Studies indicate that clinically relevant drug concentrations are typically confined to the superficial 2 to 3 mm of brain parenchyma [116,117]. As a result, these delivery methods may not facilitate effective drug penetration into the deeper regions of the brain parenchyma. On the contrary, osmotic disruption followed by IA delivery is much more effective at obtaining high drug concentrations with satisfactory drug penetration to tumor tissues. Although reports of combined use are scarce, one study evaluated the safety and efficacy of IA administration of carboplatin and melphalan combined with intrathecal topotecan chemotherapy in a 4-year-old patient with retinoblastoma metastasized to the CNS [118]. After three cycles, the authors obtained a complete response in the cerebrospinal fluid and bone marrow. Further research should focus on identifying the specific patient populations that could potentially benefit from such a combination approach [118].

### 3.9. LITT

**Figure 7:**
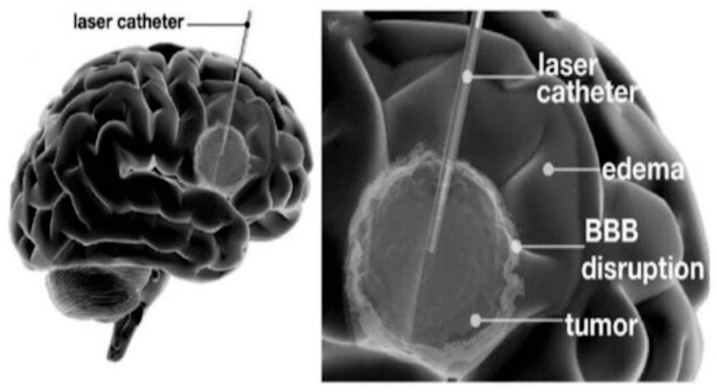
An intratumoral placement of laser catheter and brain tumor ablation. Brain tumor ablation showing post-LITT contrast enhancement consistent with LITT-related BBB disruption and LITT-related perifocal edema. Reused from Skandalakis et al. [122].

LITT involves the insertion of a stereotactically placed laser fiberoptic catheter into the brain, which utilizes thermal energy to ablate tissue surrounding the laser emission tip through coagulative necrosis (Figure 7), simultaneously exposing the peri-ablation tissue to sublethal temperatures [119,120,121,122]. Precise control of the ablation area is achieved thanks to real-time monitoring of temperature distribution through spatial and temporal MR thermometry [121]. The observation of increased BBB permeability in peri-ablation regions has led to the hypothesis that the hyperthermia induced by LITT could result in transient BBBD. Subsequent studies have supported this hypothesis by demonstrating that LITT disrupts endothelial TJs. Furthermore, electron microscopy has revealed an increase in endothelial cell transcytosis [121,122,123].

Although LITT disrupts peritumoral BBB, the exact duration of clinically relevant BBB disruption continues to be unknown.

Using a mouse model, a study reported an increased BBB/BTB permeability after 24 h following laser ablation in a mouse model, with molecules as large as human IgG (approximately 150 kDa) passing the BBB after LITT [124]. In 14 glioma patients, Leuthardt et al. found that the peak permeability occurs at one to two weeks after ablation and lasts for up to four to six weeks [120], whereas Bartlett et al. reported peaking permeability at two weeks and returning to baseline by eight weeks postoperatively in a similar cohort of patients [121]. Large cohort studies are necessary to precisely evaluate the duration of LITT induced-BBBD as well as define the size-selective characteristics of the BBBD.

To date, a phase II study of 41 patients with glioblastoma undergoing LITT combined with 20 mg/m^2^ IV doxorubicin is the only trial that evaluated the safety and efficacy of this novel technique [94]. Authors reported higher overall survival (OS) compared to historical controls treated with bevacizumab or LITT with standard salvage chemotherapy [125]. However, it remains unclear to what extent the therapeutic benefit was attributed to the cytoreductive properties of LITT and the increased uptake of doxorubicin through disrupted BBB/BTB. Likewise, this trial did not assess the spatial distribution or conduct longitudinal measurements of doxorubicin concentrations in the CSF after LITT. A more detailed understanding of doxorubicin concentrations, particularly at the tumor margin, could help determine the optimal duration and number of doxorubicin doses for achieving improved survival outcomes.

Satisfactory results coming from this trial support the combination of LITT with other chemotherapeutics; however, given that the authors reported poor CNS penetration of doxorubicin distal to the laser catheter, LITT may be a suitable method of BBBD in nodular, compact lesions rather than highly infiltrative HGGs [125].

Although the relative scarcity of studies makes any declaration of safety and efficacy impossible, ongoing clinical trials evaluating several LITT-combination therapies, including LITT combined with immune checkpoint inhibitors, such as pembrolizumab (NCT02311582) and avelumab (NCT03341806), will provide valuable answers regarding this promising method.

### 3.10. CED

Founded on the principle of fluid convection, also known as “bulk flow”, convection-enhanced delivery uses pressure gradients to facilitate the passage of therapeutic agents through tissue [126,127,128,129]. By applying continuous positive pressure through microcatheters connected to pumps, the infused chemotherapeutics are propelled and delivered throughout large volumes of brain tissue [126,127,128,129]. In comparison to diffusive delivery, the distribution of macromolecules with CED is not influenced by size and molecular weight [129,130].

CED’s device is characterized by a mostly spherical distribution of therapy at a controlled rate of 0.5 to 10 µL/min. [126,128,129,130]. Given that it does not depend on a steep concentration gradient to propel flow, CED allows for the delivery of a homogenous concentration of a chemotherapeutic throughout its volume of distribution (Vd) [129]. Unlike single injection techniques, which distribute therapy up to 5 mm from the catheter tip, CED distributes the chemotherapeutic agent up to 6 cm from the catheter tip, offering a 4000-fold increase in the volume of distribution [126,127,128,129].

Although CED allows for precise delivery of therapeutic concentrations while minimizing systemic exposure, it is an imperfect method, requiring further research and development. One of the significant drawbacks is backflow, which occurs when the delivered drug exits the targeted tissue and spreads to normal brain and CSF spaces, potentially resulting in pronounced immune activation in normal parts of the CNS and restricting dose delivery to the pathological site [127,128,129].

The exact causes behind this phenomenon are not known. However, the introduction of tapered tips and soft or porous membrane constructs into the catheter design as well as the guidelines recommending placing catheters at least 2 cm from pial surfaces and resection cavities have significantly contributed to minimizing backflow occurrences [127,128,129]. Additional strategies, including the reduction in trauma during catheter insertion, delayed initiation of infusion, and a gradual increase in infusion rates, have further enhanced the effectiveness of mitigating backflow.

CED has been also limited by the lack of visualization of the distribution, prompting researchers to consider the use of a detectable radioisotope or a surrogate tracer such as gadolinium-DPTA or gadoteridol-loaded liposomes mixed with the infusate to allow for real-time visualization of the drug distribution and therefore more precise delivery [129,131].

To date, a considerable number of clinical trials have been published, evaluating the safety and efficacy of CED in improving the penetration of traditional chemotherapeutics, conjugated toxins, liposomes, and viruses in glioma patients, reporting encouraging results, with median overall survival ranging from 16 to 58.5 weeks [129,130,132,133,134,135,136,137,138,139,140,141,142]. While the potential of CED to enhance drug penetration and achieve therapeutic concentrations has been demonstrated, the fact that only a handful of patients showed sustained improvement underscores the challenging nature of gliomas brought by their highly immunosuppressive microenvironment [129,130,132,133,134,135,136,137,138,139,140,141,142]. Likewise, CED may be less efficacious in gliomas with a rich heterogeneous network of the vasculature as it may be associated with poor drug distribution to more peripheral areas of diffuse gliomas and drug reflux [127,128,129]. The PRECISE study was the first phase III clinical trial, which compared CED of cintredekin besudotox (a recombinant protein consisting of interleukin-13 (IL-13) and a truncated form of Pseudomonas exotoxin (PE38QQR) 96 h after surgical resection with carmustine wafers placed at the time of surgery in 296 adult patients with glioblastoma multiforme at first recurrence. The trial did not demonstrate a survival difference between CED-delivered cintredekin besudotox and carmustine wafers (9.1 months vs. 8.8 months, respectively) [136]. Although the safety profile was similar in both arms, pulmonary embolism was more common in the cintredekin besudotox arm (8% vs. 1%, respectively) [136]. However, this trial has been limited by its ability to detect only benefits greater than 50% in survival, with any smaller benefits requiring a larger cohort, as well as the lack of drug distribution evaluation [97]. Moreover, 11% of patients did not fulfill inclusion criteria, and only 27% had complete resections prior to treatment. Last but not least, tumor specimens from the original surgery were not evaluated for the presence of IL13 receptors. Considering these limitations, follow-up studies have been conducted, but no improvement in PFS from 11 to 18 weeks with IL-13PE38QQR was observed [129]. However, given that the volume distribution has not been evaluated, it remains unclear whether the lack of therapeutic benefit is attributable to the delivery method or the chosen therapeutic.

CED seems to be a promising method in terms of safety and efficacy in obtaining high therapeutic drug level concentrations without inducing systemic toxicity. Future trials comparing the delivery of the same therapeutic agent between CED and the comparative arm are crucial to fully evaluate the potential of CED. Considering that CED outcomes are strongly dependent on technical aspects, such as catheter configuration and position, infusion rate, and volume, future trials should establish specific guidelines that allow for optimal CED. Once the technical aspects are refined, attention can be shifted towards identifying therapeutics that could be of greatest efficacy when delivered via CED [127,128,129].

## 4. Non-Invasive Methods of BBBD

### 4.1. Ultrasound-Mediated BBB Opening

#### High-Intensity Focused Ultrasound

The idea of using high-intensity focused ultrasound (HIFU), also referred to as focused ultrasound (FUS), along with intravenously administered micron-scale microbubbles (MBs), to temporarily disrupt BBB/BTB has gained significant traction during the last decade [143,144].

FUS utilizes acoustic waves that pass through the skull and converge at a specific focal point within the brain [143,144]. Within this focal point, the FUS beam targets intravenously injected microbubbles, which subsequently respond to the rarefactions and compressions of the applied pressure wave by oscillating [145,146]. Such oscillation has adequate energy to exert mechanical stress onto the cells, leading to the opening of TJs between ECs of the BBB [145,146]. FUS has also been shown to enhance the active transport of molecules across the BBB *through* various pathways, such as up-regulation of vesicles and carrier proteins or modulation of mechanosensitive ion channels [145,146]. Lastly, given that FUS provokes a transition from diffusive transport to convective transport in the tumor interstitial space, this method could be potentially suitable for the delivery of larger therapeutics, such as antibodies, whose transport across the BBB is largely reliant on convection [22]. Aside from BBB opening, there is a growing body of evidence pointing towards FUS’s ability to remodel brain vasculature or stimulate the development of new neurons [147].

The duration of BBB opening after the treatment varies but is generally reported to last for approximately 4–6 h before the barrier slowly returns to its normal state, with the duration and extent of BBB opening depending on the intensity, sonication time, the size and concentration of the intravenously administered microbubbles, as well as individual patient characteristics [144,148]. Higher intensity levels and longer sonication times result in more extensive BBB disruption [144,148]. The higher the intensity level, the higher the acoustic pressure, and as a result, the higher the BBB permeability [149]. As much as it may be advantageous, particularly for the delivery of therapeutics in the size range of 500–2000 kDa, higher acoustic pressure comes with inherent risks, such as large volumetric oscillations of the microbubbles and, potentially, their collapse [147,149,150]. Known as inertial cavitation, this phenomenon generates extra mechanical stresses on the capillaries in the form of micro-jets that can damage the surrounding capillary and brain parenchyma [149,150]. However, irrespective of the cavitation specifics, numerous studies attest to the safety of FUS-mediated BBBD [147,149,150].

Using a mouse model, Morse et al. developed a rapid short-pulse (RaSP) ultrasound sequence (center frequency: 1 MHz; peak-negative pressure: 0.35 MPa; pulse length: 5-cycles; pulse repetition frequency: 1.25 kHz; burst length: 10 ms; burst repetition frequency: 0.5 MHz) that effectively delivers compounds of sizes up to a hydrodynamic diameter between 8 nm (70 kDa dextran) and 11 nm (immunoglobulin) [151,152]. According to the authors, raising the acoustic pressure can expand the use of rapid short-pulses to effectively deliver agents beyond this threshold, with practically no compromise of the safety profile [151,152].

The animal studies have demonstrated that, by disrupting the BBB/BTB as well as augmenting brain tumor interstitial flow velocity and changing ‘‘per voxel” flow direction, FUS improves the penetration of drugs and multifunctional agents [153] with reports of enhanced antitumor efficacy. These findings have constituted a foundation for various clinical trials evaluating the combination of FUS with pembrolizumab in patients with brain metastases from lung cancer (NCT05317858), trastuzumab in brain metastases from breast cancer (NCT03714243), and doxorubicin in pediatric patients suffering from diffuse intrinsic pontine gliomas (NCT05630209, NCT05615623).

Initially, FUS was guided by diagnostic ultrasound imaging, which has no capacity to determine the real-time temperature and is associated with poor guidance accuracy.

In order to provide a controlled and focal disruption, Hynynen et al. recommended combining FUS with high-resolution three-dimensional MRI as its exceptional spatial control over the treatment field could allow operators to ensure accurate placement of the focal spot at the planned treatment site [154]. MRI identifies the presonication volume, while postsonication temperature is measured by proton resonance frequency shift by means of fast gradient-echo sequences [154,155]. Lastly, the ablated volume is identified with T2-weighted fast spin-echo sequences [154,155].

A phase I, single-arm, open-label study involving five patients with high-grade glioma was the first to investigate the combination of magnetic resonance-guided focused ultrasound (MRgFUS) and chemotherapy [156]. The patients received either liposomal doxorubicin (n = 1) or temozolomide (n = 4) one day before their scheduled surgical resection. During the procedure, MRgFUS was used to disrupt the BBB, and the disruption was observed to last for up to 20 h [156]. The study findings revealed that the concentration of chemotherapy was higher in the tissue where BBB disruption occurred in comparison to the non-BBB disrupted tissue [156].

Studies conducted on mice have demonstrated that FUS-induced opening of the BBB releases intraparenchymal substances, including brain-specific proteins and mRNA [157]. These findings have been confirmed by a recent trial of nine glioblastoma patients undergoing adjuvant chemotherapy with MRgFUS. The resulting BBB opening enhanced circulating brain-derived proteins, neuron-derived extracellular vesicles, and cell-free DNA, showing that MRgFUS could be potentially used to help assay changes in the tumor microenvironment that might predict recurrence or a need for alternative therapy [157].

Recently, studies have been aiming to optimize the delivery of therapeutic agents across BBB and into the brain parenchyma by combining FUS with Photoacoustic (PA) imaging, which is a novel imaging technique integrating optical excitation with ultrasound detection to enable the visualization of deep brain tissues with high resolution and depth penetration [158,159]. Studies have found that integrating PA with FUS allows to overcome the skull-led light and ultrasound attenuation [158,159]. Zhang et al. integrated ultrasmall Cu_2−x_Se nanoparticles, known for their excellent contrast-enhancing properties in PA imaging, with doxorubicin (DOX)-loaded organic–inorganic hybrid hollow mesoporous organosilica nanoparticles (HMONs) [159]. Using Focused Ultrasound (FUS) in mice, this nanosystem was successfully delivered into orthotopic brain tumors. Subsequently, the nanosystem efficiently penetrated the tumor tissue, exerting an antitumor effect, with the entire process being visualized in real-time with PA [159].

### 4.2. Low-Intensity Pulsed Ultrasound

Low-intensity pulsed ultrasound with concomitant administration of intravenous microbubbles (LIPU-MB) is another ultrasound method used to transiently open the BBB/BTB [160]. The principle is the same as with FUS, i.e., oscillating microbubbles upon stimulation by ultrasound provoke mechanical stress on the endothelial wall, resulting in BBB opening [160]. However, LIPU utilizes lower-intensity waves (<3 W/cm^2^) distributed over a broader area in comparison to FUS (≥3 W/cm^2^) [161]. Moreover, LIPU functions as a skull-implantable ultrasound device fixed to the bone using standard surgical screws in comparison to FUS, which is non-implantable and applied externally to the body [160]. Lastly, LIPU does not involve MRI guidance. Given that the level of BBB disruption is dependent on the intensity of ultrasound, LIPU should theoretically induce a less sustained or shorter BBB disruption than FUS. Nevertheless, there are no comparative studies directly comparing the degree of BBB opening obtained by these two modalities.

Recently, a phase 1 clinical trial (NCT04528680) involved 17 patients with recurrent glioblastoma who underwent tumor resection and LIPU-MB using a skull-implantable ultrasound device (SonoCloud-9 [SC9]; Carthera, Lyon, France) with intravenous albumin-bound paclitaxel infusion conducted every 3 weeks for up to six cycles [160]. Overall, six dose levels of albumin-bound paclitaxel (40 mg/m^2^, 80 mg/m^2^, 135 mg/m^2^, 175 mg/m^2^, 215 mg/m^2^, and 260 mg/m^2^) were evaluated [160]. Pharmacokinetic analyses showed that LIPU-MB led to a 3.7-times increase in the mean brain parenchymal concentrations of albumin-bound paclitaxel when compared to the non-sonicated brain, with no signs of neurologic deficits attributed to LIPU-MB [160]. Analyzing a subgroup of patients who received carboplatin as part of a similar trial (NCT03744026), the authors found a 5.9-times increase in the mean brain parenchymal concentrations in a sonicated brain [160]. These highly encouraging results will be further evaluated in a phase 2 study combining LIPU-MB with albumin-bound paclitaxel plus carboplatin (NCT04528680).

Ultrasound-based therapeutic techniques have been shown to safely and transiently increase the permeability of the BBB in small brain regions, allowing for closely controlled delivery of therapeutic agents [160,161,162]. It would be recommended to test the synergistic effect of ultrasound-based therapy with IA delivery of chemotherapeutics and compare these outcomes to osmotic BBBD alone. 

### 4.3. TTFields

TTFields are an emerging therapeutic approach involving the application of low-intensity (1–3 V/cm), intermediate-frequency (100–300 kHz), alternating electric fields delivered via transducer arrays applied to the scalp [163]. Initial studies found that TTFields can induce mitotic arrest and apoptosis in rapidly dividing cells, offering efficacy comparable to chemotherapy regimens but with lower toxicity [163,164]. However, subsequent investigations showed that the anticancer effect of TTFields is attributable to an array of various biological mechanisms, including anti-migratory effect, DNA repair and stimulation of autophagy, as well as antitumor immunity [164]. More importantly, research has found that TTFields can enhance membrane permeability in glioma cells by increasing the number and size of membrane pores, with an average hole size of 240.6 ± 91.7 nm^2^ in TTFields-treated cells in comparison with 129.8 ± 31.9 nm^2^ in untreated cells [163]. This increase in permeability allowed for improved uptake of membrane-associating reagents with a size of up to 20–50 kDa into glioma cells, with the permeability normalizing within 24 h of ceasing TTFields treatment [163]. Crucially, these alterations seem to be exclusive to cancer cells, as no shifts in the membrane composition of healthy human fibroblast cells were detected [163]. The mechanism behind the increased permeability of BBB is attributable to transient disruption of the localization of tight-junction proteins such as claudin 5 and ZO-1 [163,164,165].

A phase III clinical trial of 637 randomized glioblastoma patients aimed to compare the effects of TTFields with maintenance temozolomide vs. maintenance temozolomide alone [166]. The study reported that TTFields combined with temozolomide were associated with a longer median progression-free survival from the time of randomization (6.7 months vs. 4.0 months) and longer median overall survival from randomization (20.9 months vs. 16.0 months, respectively) [166]. There were no significant differences in the rates of systemic adverse effects between the two treatment groups (48% vs. 44%) [166]. Given the well-tolerated nature of TTFields and their minimal impact on the patient’s quality of life, even considering the visibility of the device, the substantial benefits of this novel therapy far outweigh any potential drawbacks, supporting its addition to the standard chemotherapeutic regimen [166]. However, since this trial did not assess BBB opening, it remains unclear whether the therapeutic benefit is primarily due to the enhanced uptake of temozolomide facilitated by BBB disruption or if it arises from the synergistic effect of various biological mechanisms exerted by the TTFields. Further research is crucial to outline how different TTFields properties translate into tangible clinical improvements.

TTFields, along with other reviewed methods of BBBD, have been summarized in Table 1.

## 5. Future Directions

BBB/BTB structural integrity is highly heterogeneous within metastatic lesions and different tumor types [22]. Assessing and quantifying this heterogeneity before and after therapy through PET or dynamic contrast-enhanced (DCE) MRI could help identify optimal treatment windows and thereby support the selection of adequate methods of improving drug delivery across the BBB/BTB [22]. Continued research into the pharmacological kinetics of the BBB/BTB will permit determining the most effective dose for a specific agent for osmotic BBBD coupled with IA administration [73,97]. Similarly, it is essential to establish new robust pharmacokinetic models and improve our understanding of the therapeutic distribution as hydrodynamic factors, such as the background blood flow, injection characteristics, and vascular geometry, have a significant role in determining tissue concentrations after osmotic BBBD with IA injection [73,97]. This should also include the growing field of nanotechnology since smaller particles are subjected to substantially smaller hydrodynamic forces and thereby may require different injection velocities or catheter shapes [73,99].

Thus far, osmotic BBBD with IA administration has primarily focused on delivering established chemotherapies, such as carboplatin or nimustin, with novel agents, such as bevacizumab or cetuximab, having been studied only during the recent decade [73,97]. However, combining osmotic BBBD with IA delivery of mesenchymal stem cells that are used as vehicles for drugs due to their significant tumor tropic properties and the ability to traverse BBB endothelial cells through either the paracellular or transcellular routes holds significant promise for advancing brain tumor treatment [73]. Similarly, osmotic BBBD with IA delivery should be evaluated with immunostimulatory molecules, nanoparticles, and antiproliferative proteins. To support the evaluation of different therapeutic agents, it is crucial to come up with tumor-bearing animal models with a cerebrovascular system similar to that of humans and a possibility to accommodate clinical catheters [99].

Finally, it is imperative to conduct phase 3 clinical trials or long-term prospective studies to answer various safety questions, including the effect of repetitive osmotic disruptions on the BBB structure, as to date only single-phase 1/phase 2 studies reported outcomes so far [73,97]

Given that intrathecal/intraventricular administration is an inadequate method for overcoming the BBB/BTB and improving drug concentrations in deeply situated tumors, studies should test its combined use with other methods, such as osmotic BBBD with IA administration, and evaluate which patient population could potentially benefit from such an integrated approach.

LITT is an emerging technique allowing for minimally invasive cytoreduction of brain tumors. Although studies combining LITT with chemotherapy hypothesize that improved survival for patients with recurrent glioblastoma is attributable to the LITT-induced BBBD rather than local control, little is known about the mechanisms underlying this effect [120]. Likewise, the exact duration of clinically relevant BBB disruption or its degree remains unknown. Research into this area, together with well-designed randomized clinical trials, is crucial to bring this novel method to clinical use.

Although CED is a promising method, it needs further improvement before it can be fully introduced clinically. Given that the currently used externalized catheters attached to pumps may increase the risk of infection as the length of therapy extends, semi-permanent, refillable pump-catheter implants permitting continued regional infusion of therapeutic agents into the brain parenchyma for extended periods are in development [129]. Moreover, considering the possibility of refilling the pump in the outpatient setting, these pump-catheter implants facilitate repetitive, cyclical infusions, essential to target tumor cells that may not be actively dividing at the time of infusion [129]. Additionally, research should focus on important factors, such as catheter design, number of catheters, placement of catheters, infusion rate, duration of infusion, and methods of monitoring drug distribution [129]. Subsequently, standardized guidelines should be introduced to guarantee the reproducibility of outcomes.

Proof-of-concept studies have demonstrated safe, temporary BBB disruption in tumor and peritumoral tissue following MRIgFUS with IV-administered microbubbles [156]. Future trials should reproduce these results in a greater number of patients and evaluate different sonication parameters in order to tailor BBB disruption to various brain tissues, tumors, and otherwise [156]. Considering reports of issues with reproducibility of FUS procedures, it is imperative to establish standardized guidelines to ensure the consistency and replicability of results. Last but not least, considering that an integrated approach to BBBD may yield better results, MRIgFUS should be evaluated in combination with SIACI.

Demonstrated in a large group of patients, LIPU-MB can safely open the BBB to allow for repeated penetration of large drugs into the brain. However, significant pharmacokinetic questions continue to be unanswered, such as the temporal and spatial dynamics of drug accumulation, dispersion, and clearance in the human brain [160]. Similarly, the characterization of the effect of LIPU-MB on drug concentrations in tumor tissue has not been tested [160].

TTFields exert an array of biological effects, with transient increased BBB permeability being one of them [166]. Given that it is unclear whether the reported improved survival for patients with glioblastoma after TTFields and temozolomide are attributable to the BBBD or its anti-mitotic properties, studies should clearly outline TTFields’ potential to disrupt the BBB, i.e., the extent of BBBD and its duration. Similarly, investigating which specific drugs can effectively penetrate the BBB following TTFields is essential.

Due to the complex and heterogeneous nature of brain tumors, it is unlikely that a single therapeutic agent will be sufficient to address the diverse aspects of their pathogenesis. It is imperative, therefore, to evaluate various combinations of therapeutic agents, leveraging their synergistic effects to potentially yield tangible clinical benefits. Subsequently, studies should evaluate which method of overcoming the BBB/BTB is most suitable for the delivery of a particular combination.

## 6. Conclusions

Although our understanding of the BBB and BTB structures has made significant strides in recent decades, it is crucial to focus on elucidating how these alterations translate into functional differences and thereby influence drug distribution to the CNS.

Various safe and effective techniques for opening the blood–brain barrier exist, and the selection of the optimal method will depend on the specific application and individual patient characteristics.

Osmotic BBBD coupled with IA administration is an old and well-established concept allowing the overcoming of the challenges of delivering many therapeutic agents across the BBB/BTB, particularly targeted biological therapeutics. Not only is it highly effective at obtaining local, clinically relevant concentrations of the administered drug to large brain areas, but with adequate selection of cerebral vessels, real-time guidance, implementation of flow arrest techniques, and pulsatile injections, it can be highly safe in experienced hands, with severe long-term complications being uncommon. With constant refinement of the technique, improved understanding of therapeutic distribution, and evaluation of novel agents, such as immunotherapeutics or gene therapy, this method may have profound implications for the future treatment of brain tumors.

Given that intrathecal/intraventricular administration often falls short in achieving clinically relevant drug concentrations for deeply situated tumors, it is advisable to restrict its use to pathologies within 2 to 3 mm of the brain parenchyma’s surface. Alternatively, in the case of pathologies extending to deeper parts of the brain parenchyma, it could be supported by osmotic BBBD with IA delivery.

The field of CED has been extensively studied since its conception; however, the heterogeneity of findings coming from preclinical and clinical studies has significantly limited the evaluation of its potential to enhance anti-tumor responses. As much as CED remains a promising method, allowing for homogenous and safe drug delivery to the tumor, technical refinement and the establishment of standardized guidelines based on conclusions from well-designed clinical trials are crucial to optimize this method.

While LITT’s potential to effectively ablate local brain tumors is backed by a growing body of literature, its ability to disrupt the BBB continues to be poorly understood. Preclinical studies provide substantial proof that LITT disrupts the BBB; however, its clinical relevance is unknown. The hypothesis, based on reports of improved survival in patients who underwent LITT with immunotherapy, that LITT improves the efficacy of immunotherapy through BBBD has hitherto not been supported by any direct evidence.

MRIgFUS with intravenous administration of microbubbles is a precise and safe method that allows the disruption of the BBB for longer periods of time than osmotic BBBD, making it a potentially suitable method for drug delivery to small brain regions. Similarly, given that long-term studies have highlighted the ability of LIPU-MB to allow for safe and repeated penetration of cytotoxic drugs into the brain, it could be a potentially attractive alternative to MRIgFUS. Comparison of these two ultrasound technologies with respect to their efficacy in improving drug penetration across the BBB/BTB warrants further research.

While the antimitotic effect of TTFields supporting the efficacy of chemotherapy in inducing cell cycle arrest is well established, its potential ability to disrupt the BBB has only been evaluated in preclinical settings. Consequently, it is unknown whether TTFields may allow for improved drug penetration in brain tumor patients, highlighting the need for further investigation.

Osmotic BBBD, intrathecal/intraventricular administration, and CED are well-studied methods in comparison to emerging therapies, such as LITT, ultrasound approaches, and TTFields. Considering the currently available evidence, it is impossible to predict whether these novel methods of overcoming BBB will become fully integrated clinically.

The future of manipulating the BBB for brain tumor treatment may not hinge on a singular method but rather on the synergistic combination of several approaches. Precise opening of the BBB using osmotic disruption or focused ultrasound, followed by the selective IA delivery of drugs supported by real-time monitoring of drug delivery, could be a viable option for obtaining local, clinically relevant concentrations of the administered drug while reducing systemic toxicity.

The successful treatment of brain tumors will depend on a collaborative and concerted effort between researchers, pharmacologists, and industry partners. The seamless integration of the emerging field of precision medicine with the ever-evolving landscape of medical technologies will be pivotal in shaping the next frontier of effective brain tumor treatments.

### Limitations

This scoping review is limited by the heterogeneity of studies on the BBB, including varying experimental designs, sample sizes, different endpoints to assess BBB disruption or the diversity of animal models used in preclinical studies.

## Figures and Tables

**Table 1:** Methods of overcoming/disrupting BBB in drug delivery to the CNS. The table lists the duration of BBB disruption, underlying mechanisms and advantages as well as disadvantages of reviewed methods of BBB disruption.

Methods of Overcoming/Disrupting BBB in Drug Delivery to the CNS				
Method	Mechanism of BBBD	Duration of BBB Disruption	Advantages	Disadvantages
Invasive methods of overcoming/disrupting BBB				
OBBBD	Infusion of an osmotic agent, e.g., mannitol leads to dehydration of endothelial cells and subsequent disruption of the tight junctions between endothelial cells of the BBB.	The maximum effect in humans lasts up to 40 min and returns to baseline levels only after 6 to 8 h [105] ^.	may increase drug exposure by up to 100-fold following IA delivery. allowing for the delivery of large therapeutic molecules, such as bevacizumab (149 kDa) or cetuximab (approximately 152 kDa) [93,103]. the possibility of real-time monitoring. depending on the catheter positioning, it can lead to selective or extensive BBBD.	requires general anesthesia. the high variability of the area of BBBD. minor complications, such as seizures, asymptomatic strokes, and transient neurological decline (7.53% of procedures) [101]. major complications, including myocardial infarction, cervical cord injury, symptomatic stroke, and death (0.70% of procedures) [101].
Intrathecal/intraventricular administration	NA	NA	allows for the delivery of large molecules. facilitates multiple drug injections.	obtains clinically relevant drug concentrations confined to the superficial 2 to 3 mm of brain parenchyma beyond the subarachnoid space due to interstitial fluid pressure [116,117]. invasive. potential complications, such as drug-induced aseptic meningitis (DIAM) and infection of the reservoir through which the agents are administered.
LITT	Disruption of endothelial tight junctions and increases endothelial cell transcytosis.	‡ the peak permeability of the BBB occurs one to two weeks after LITT and returns to baseline by eight weeks postoperatively.	precise BBBD. allows for the penetration of molecules as large as human IgG (approximately 150 kDa) [124].	invasive, must be performed under general anesthesia. poor drug penetration in CNS distal to the laser catheter [125]. potential for arterial injury, seizures, transient or permanent neurologic deficit, cerebrospinal fluid leak, and infection. poorly studied.
CED	A stereotactically placed catheter is connected to an infusion pump that generates the pressure gradient to facilitate the controlled and direct infusion of therapeutic agents into the extracellular space of the brain.	NA	allows for a predictable delivery of a homogenous concentration of a chemotherapeutic. allows for direct access to the tumor bed. the concentration gradient of the infused agent tends to fall off sharply at the periphery of the targeted region, reducing the risk of brain toxicity. distributes the chemotherapeutic agent up to 6 cm from the catheter tip, offering a 4000-fold increase in the volume of distribution [126,127,128,129].	backflow. white matter edema. the loss of infusate to circulation in the case of highly vascularized active tumors.
Non-invasive methods of overcoming/disrupting BBB				
FUS-MB	FUS-triggered oscillation of microbubbles opens tight junctions in the BBB. Up-regulation of vesicles and carrier proteins or modulation of mechanosensitive ion channels.	20 h * [156].	noninvasive, can be performed with mild sedation. MRI guidance allows for specific delivery to the targeted small brain area. facilitates the delivery of many different classes of compounds, particularly larger ones (bevacizumab, liposomal doxorubicin, albumin-bound paclitaxel plus carboplatin).	expensive. microhemorrhages and neuroinflammation. the need for constant adjustment of microbubble dosing to prevent damage to the endothelium [156].
LIPU-MB	Microbubbles oscillate upon stimulation by ultrasound, producing mechanical stress on the endothelial wall that disrupts the BBB.	most BBB integrity is restored within 1 h after LIPU-MB [160].	allows for repeated drug penetration to the brain parenchyma (albumin-bound paclitaxel plus carboplatin) [160]. achieves BBBD in deep, critical brain structures such as the thalamus and basal ganglia [160].	targets small brain volume (approximately 53 mL.) [160]. fixed field of sonication. the need for percutaneous connection of the device, limiting the frequency of LIPU-MB. the device has to be implanted.
TTFields	Disruption of the localization of tight-junction proteins such as claudin 5 and ZO-1.	The BBB permeability normalizes within 24 h of ceasing TTFields treatment [164].	allows for improved uptake of membrane-associating reagents with a size of up to 20–50 kDa into glioma cells [164]. non-invasive. the enhanced permeability seems to be limited to cancer cells. it may also exert an anticancer effect through various biological mechanisms. non-invasive.	emerging method requiring further evaluation. irritant or allergic contact dermatitis at the site of transducer array attachment [166]. a total monthly cost of around $21,000 [164].

OBBBD—osmotic blood–brain barrier disruption ^ May differ depending on the used anesthetic agents, the duration and rate of infusion of mannitol, or the physiological parameters such as blood pressure, heart rate, and PaCO_2_, BBBD—blood–brain barrier disruption, LITT—laser interstitial thermal therapy history, CED—Convection-Enhanced Delivery, FUS-MB—focused ultrasound with concomitant administration of intravenous microbubbles, LIPU-MB—low-intensity pulsed ultrasound with concomitant administration of intravenous microbubbles, TTFields—tumor-treating fields, ‡ the exact duration of clinically relevant BBB disruption continues to be unknown. * depending on the intensity, sonication time, size, and concentration of the intravenously administered microbubbles, as well as individual patient characteristics, NA—not applicable.
